# Finding fault: Causality and counterfactuals in group attributions

**DOI:** 10.1016/j.cognition.2012.07.014

**Published:** 2012-12

**Authors:** Ro’i Zultan, Tobias Gerstenberg, David A. Lagnado

**Affiliations:** a Department of Economics, Ben-Gurion University of the Negev, Israel; b Cognitive, Perceptual and Brain Sciences Department, University College London, United Kingdom

**Keywords:** Responsibility, Attribution, Counterfactuals, Causality, Groups

## Abstract

Attributions of responsibility play a critical role in many group interactions. This paper explores the role of causal and counterfactual reasoning in blame attributions in groups. We develop a general framework that builds on the notion of pivotality: an agent is pivotal if she could have changed the group outcome by acting differently. In three experiments we test successive refinements of this notion – whether an agent is pivotal in close possible situations and the number of paths to achieve pivotality. In order to discriminate between potential models, we introduced group tasks with asymmetric structures. Some group members were complements (for the two to contribute to the group outcome it was necessary that both succeed) whereas others were substitutes (for the two to contribute to the group outcome it was sufficient that one succeeds). Across all three experiments we found that people’s attributions were sensitive to the number of paths to pivotality. In particular, an agent incurred more blame for a team loss in the presence of a successful complementary peer than in the presence of a successful substitute.

## Graphical abstract

**Graphical abstract:**

**Figure:**
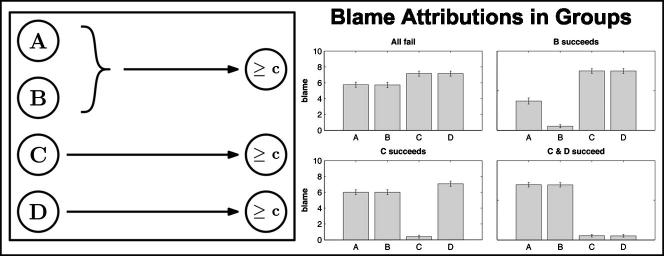

**Highlights:**

► We develop a general framework of responsibility attributions in groups. ► Three experiments show that blame varies with the relationships between agents. ► Agents are blamed more if their complement succeeds rather than their substitute. ► Blame attributions are sensitive to ways in which an agent can make a difference. ► The importance of understanding responsibility attributions in groups is discussed.

We have come to think of the actual as one among many possible worlds. We need to repaint that picture. All possible worlds lie within the actual one. (Goodman, 1983, p. 57)

## 1 Introduction

Your football team has just lost an important match after the goalkeeper failed to save an easy shot. How much is the goalkeeper to blame for the team’s loss? Does it matter that the final score was 0–2, so that the goal in question did not affect the outcome of the match? Is the goalkeeper’s blame moderated by the fact that the forward in your team missed a penalty kick? Would it make a difference if you could know that the penalty kick would have been saved by the other team’s goalkeeper anyway?

Team sports is a commonplace context in which blame (and credit) is attributed to individuals for their team’s outcome. Such attributions are also prevalent and carry serious implications in contexts as diverse as business and criminal law (e.g., Hart, 2008).

The potential importance of responsibility attributions in general has lead to the development of a substantial literature looking at the psychological processes behind responsibility attributions (Alicke, 2000; Lagnado & Channon, 2008; Shaver, 1985; Weiner, 1995). Nonetheless, little research has been conducted on responsibility attributions in team environments. Specifically, we do not yet have a good understanding of how the performance of one team member moderates the responsibility of her partners.

In this paper we consider different ways to model responsibility attributions in a simple team environment. We identify behavioral principles revealed in three experiments designed to distinguish between the different models and develop a new model, which extends the structural model proposed by Chockler and Halpern (2004) and studied by Gerstenberg and Lagnado (2010) to capture these principles.

According to Chockler and Halpern’s structural model, there is a close relationship between causality, counterfactuals and attributions of responsibility (see also Petrocelli, Percy, Sherman, & Tormala, 2011). An individual is deemed responsible if she was pivotal in the actual situation, whereby pivotal means that the (team) outcome counterfactually depends on her action. Hence, a person is pivotal for a loss if she would have made the team win had she performed better and, conversely, a person is pivotal for a win if she would have made the team lose had she performed worse.

While the intuition is strong that a person carries responsibility in a situation in which she would have made a difference to the outcome, it is less clear whether a person can be held responsible in a situation in which she would not have changed the outcome. According to a simple pivotality model the answer is negative: someone cannot be responsible for an outcome that would have occurred irrespective of their action (see below). Chockler and Halpern (2004), however, propose that responsibility comes in degrees. Responsibility attributions are determined by the number of changes that are required to be made in the actual situation in order to create a counterfactual situation in which the target individual *would have been pivotal* for the team outcome.

For example, the forward in the opening example is not pivotal; had he scored the penalty shot, the team would still have lost the match. Nevertheless, he would have been pivotal in the counterfactual situation in which the other team scored only one goal. The model takes this observation into account, and attributes the forward partial responsibility for the loss. Hence, a person’s responsibility is not only determined by whether her contribution made a difference in the actual situation but also by whether her contribution would have made a difference in other possible situations. In general, a person’s responsibility decreases with the number of changes that would be necessary to make her pivotal vis-á-vis the outcome.

Initial validation for the use of causal models and counterfactual considerations in responsibility attributions in team environments was provided by Gerstenberg and Lagnado (2010). In their experiment, participants form a team with three virtual players. Each player performs an individual task and the team’s outcome (win or loss) is determined as a function of the individual outcomes. The participants are then asked to attribute either credit for a team win or blame for a team loss to each of the players.^1^ Gerstenberg and Lagnado (2010) varied the way in which individual scores were combined to determine the team score. In the *sum condition*, individual scores combined additively. In the *min condition*, the team’s performance equalled the performance of the weakest player. Finally, in the *max condition* the best player in the team determined the team’s outcome. Importantly, these different causal structures have implications about the situations in which players are pivotal and how many changes would be required to render them pivotal. Gerstenberg and Lagnado (2010) found that the observed attributions were strongly correlated with the predictions of Chockler and Halpern’s (2004) model.

Nevertheless, the way in which the experiment was designed leaves room for alternative explanations. The players’ roles in the different team games that were used were always identical. For example, Chockler and Halpern’s (2004) model predicts that responsibility attributions to each individual group member in the *min condition* decrease with the number of players who failed their task. If only one player failed, he is pivotal for the loss and hence fully responsible. However, for each additional player who failed in their task, one change would be required to render the target player pivotal and responsibility attributions are predicted to decrease accordingly.

This prediction coincides with the predictions of two other, non-causal explanations. First, the principle of *diffusion of responsibility* (Darley & Latane, 1968; Wallach, Kogan, & Bem, 1964) dictates that an individual’s responsibility decreases the more people she shared it with, independent of the exact causal structure of the situation. Second, as all team members perform the same task, their performance is indicative of the task difficulty. Thus, the observed pattern can arise from a simple principle stating that one incurs less blame for failing in a difficult task compared to an easy task. Once again, the causal structure plays no role in the responsibility attributions, as responsibility is determined according to *relative performance*.

Our experiments are specifically designed to create causally asymmetric team structures in order to ascertain the roles of causal structure and counterfactual thinking in responsibility attributions, and to disentangle the alternative explanations described above. The experiments present participants with a scenario in which a team has lost its challenge and ask for attributions of blame to the team members, given their individual contributions to the team outcome. The experimental paradigm is designed to focus on the issues at hand, abstracting from additional features that are important in many natural examples. In particular, we exclude any elements that distinguish blame from the more general construct of responsibility such as epistemic states and intentions (Chockler & Halpern, 2004). In the next section, we present the general framework and four models of responsibility before proceeding to describe the experiments.

## 2 Theoretical analysis

For simplicity, we restrict our attention to team challenges in which individuals perform independent tasks and both the individual and team outcomes are binary. We consider a team with *n* agents, each performing an individual task. The outcome of agent *i* is denoted by *o*_*i*_ ∈ {0, 1}, with 0 = failure and 1 = success. The team outcome *t* is determined by a team function *t* = *f*(*o*_1_, *o*_2_, … , *o*_*n*_) ∈ {0, 1}, with 0 = loss and 1 = win. The function *f* is weakly increasing in *o*_*i*_, i.e., the team outcome cannot benefit from a *failure* of a team member, and similarly cannot be harmed by any of the team members *succeeding*.

As we show in the following, this basic framework is rich enough to capture the principles of simple causality, counterfactual causality, and diffusion of responsibility. We consider several models that differ in how they take into account peer performance and causal relationships when assigning responsibility to any one team member.

### 2.1 Simple responsibility (SimResp)

As a benchmark we consider a model, which ignores both peer performance and the causal structure. The model simply assigns a responsibility of 1 if the individual and team outcome are aligned, and 0 otherwise. In other words, if the team lost, then all the team members who have failed their individual task receive blame, and if the team won, all the team members who have succeeded receive credit.

### 2.2 Diffusion of responsibility (DiffResp)

The diffusion of responsibility model also ignores the causal relationships but takes into account peer performance as it divides the responsibility equally between all individuals who are assigned full responsibility by SimResp. The model can be interpreted as a normalized version of SimResp, in which the total responsibility sums up to exactly 1.

### 2.3 Simple pivotality (SimPiv)

The simple pivotality model refines SimResp by imposing a further condition on responsibility, namely, the model assigns a responsibility of 1 if and only if the individual and team outcomes are aligned *and* the individual is pivotal. That is, blame is only assigned to team members who failed but could have made their team win had they succeeded, given the performance of their peers.

### 2.4 Counterfactual pivotality (CFPiv)

The counterfactual pivotality model is equivalent to Gerstenberg and Lagnado’s (2010) structural model, which is derived from Chockler and Halpern’s (2004) general model of responsibility. Similar to SimPiv, CFPiv assigns a responsibility of 1 to individuals who are pivotal. The two models differ with regard to individuals who are not pivotal, but whose outcome is aligned with their team’s outcome.

In this case, the individual can be made pivotal by considering a counterfactual situation in which the individual outcomes (success/fail) of the other team members are changed. Let *N* be the number of the changes required to make the target player pivotal.^2^ The CFPiv responsibility is defined to be 1/(*N* + 1). Consider, for example, the hypothetical case where two of the authors find the same mistake in this paper. Since the mistake would have been corrected if only one of us found it, none of us was pivotal for eliminating the mistake. However, each could become pivotal through making one change to the actual situation, namely if the other had not detected the mistake. The CFPiv model therefore assigns responsibility of 1/(1 + 1) = 0.5 to each author.^3^

## 3 Experiment 1

The three models we compare to the benchmark SimResp all take into account peer performance when attributing responsibility to any team member. However, the models differ in the exact way they achieve this. As mentioned in the introduction, when the team members are symmetric with regard to their effect on the team outcome, the three models make similar qualitative predictions. To see this, consider a situation with three team members, Alice, Bob and Chuck. The causal structure is such that each team member has to succeed in their individual task in order for the team to win their challenge, that is, *t* = *min*(*o*_*Alice*_, *o*_*Bob*_, *o*_*Chuck*_). How much blame would Alice be predicted to receive in a situation in which all three failed compared to a situation in which Bob succeeded in his task? According to DiffResp, Alice receives more blame in the latter case (1/2 compared to 1/3), as the total blame is shared by fewer people. According to SimPiv, Alice would not receive any blame in either situation because she is neither pivotal in the situation in which all players failed nor when Bob succeed. Finally, CFPiv predicts that Alice’s blame increases the fewer changes are necessary to render her pivotal. In fact, it makes the same predictions as DiffResp. In the situation in which all failed, two changes are necessary to render Alice pivotal (i.e. changing *o*_*Bob*_ *and o*_*Chuck*_ from 0 to 1). In the situation in which Bob succeeded only one change is required (i.e. changing *o*_*Chuck*_ from 0 to 1). Hence, *N* = 2 in the former and *N* = 1 in the latter situation and Alice’s blame is predicted to increase from 1/3 to 1/2.

We see that, in order to make a sharp distinction between the predictions of the different models, an asymmetric structure is required. More specifically, we wish to test the qualitative prediction of the CFPiv model that the effect of one team member’s success on the blame attributed to another team member depends on the relationship between the two. If the two are *substitutes*, such that the success of one of them makes the success of the other unnecessary for the team winning, each team member must fail in order for the other to be pivotal, *t* = *max*(*o*_1_, *o*_2_). Therefore the success of one reduces the blame assigned to the other. Conversely, if the two team members are *complementary*, so that in order for one to be pivotal the other must succeed, the success of one increases the blame assigned to the other, *t* = *min*(*o*_1_, *o*_2_).

**Fig. 1:**
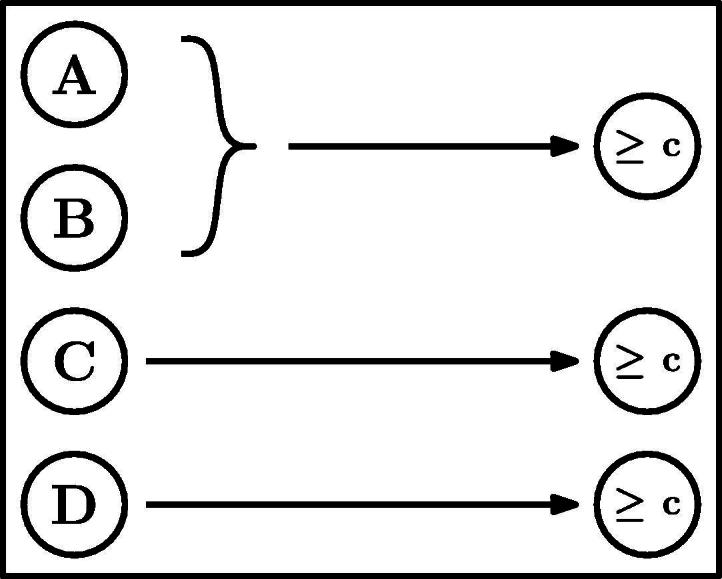
Team challenge for Experiments 1 and 2. For the team to win, *A* or *B* as well as both *C* and *D* must pass the success criterion ⩾ c. *A* is a substitute of *B* and a complement of *C*.

The team challenge we employ in Experiments 1 and 2 includes four players. Player *A* is our target player, and is a substitute of player *B* and complementary to player *C*. In line with the analysis above, we manipulate the individual outcomes of *B* and *C*, and measure the blame assigned to player *A*. To these three players we add a fourth team member, *D*, who must succeed in order for the team to win. The role of player *D* is to make it possible to manipulate the individual outcomes of players *B* and *C* without making *A* pivotal as a result. Hence, the causal structure is defined by the team function *t* = *min*(*max*(*o*_*A*_, *o*_*B*_), *o*_*C*_, *o*_*D*_). Fig. 1 shows a graphical representation of the team challenge.

**Fig. 2:**
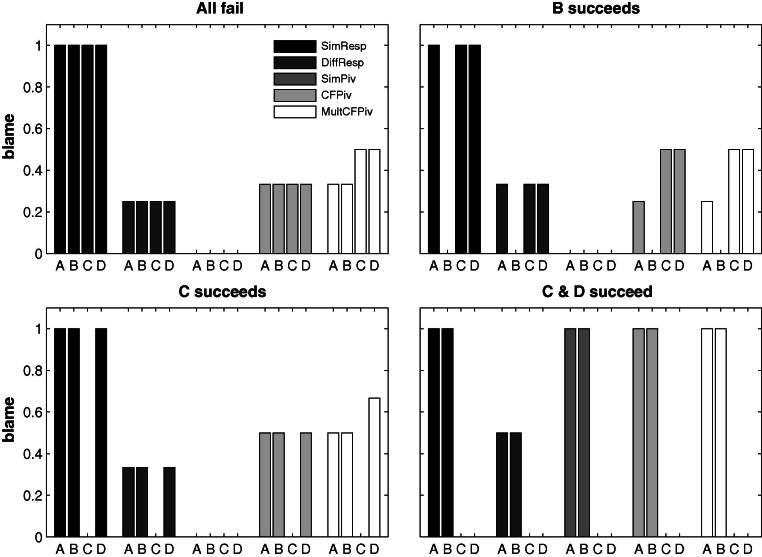
Predictions of the different models for the situations used in Experiments 1 and 2. The MultCFPiv model will be discussed below.

We compare the blame attributed to *A* in three within-subjects conditions. We test whether *A*’s blame changes when either only *B* or only *C* succeeds, compared to a baseline in which all four team members fail. Both SimResp and SimPiv predict no change in the blame attributed to *A* between the three conditions. SimResp always predicts that *A* will be blamed and SimPiv that *A* will not be blamed. Diffusion of responsibility predicts that *A*’s blame will be reduced by the same amount compared to the baseline independent of whether *B* or *C* succeeded. CFPiv is the only model predicting a difference between the two conditions in which one team member succeeds. The blame assigned to *A* should decrease with the success of his substitute *B*, and increase with the success of his complement *C*. Fig. 2 summarizes the predictions by the different models.

### 3.1 Method

#### 3.1.1 Participants

Eighty-three education undergraduate students from The Hebrew University of Jerusalem were recruited at the end of class and participated for course credit.

#### 3.1.2 Materials and procedure

All participants received identical forms that included the scenario depicted in Table 1.^4^ Each question was followed by four 7-points Likert scales. Each scale was labeled by a name (‘Oren’, ‘Benni’, ‘Gidi’, and ‘Doron’), with the end points of the scales labeled as ‘not at all’ and ‘very much’. Question 1 was presented below the scenario, whereas Questions 2–4 were presented on the back of the page with their order counterbalanced between participants. Since no effect was found for the order of presentation the responses were aggregated across orders. Participants were instructed to respond to Question 1 before turning the page and not to change their response after reading the subsequent questions.^5^

### 3.2 Results and discussion

**Fig. 3:**
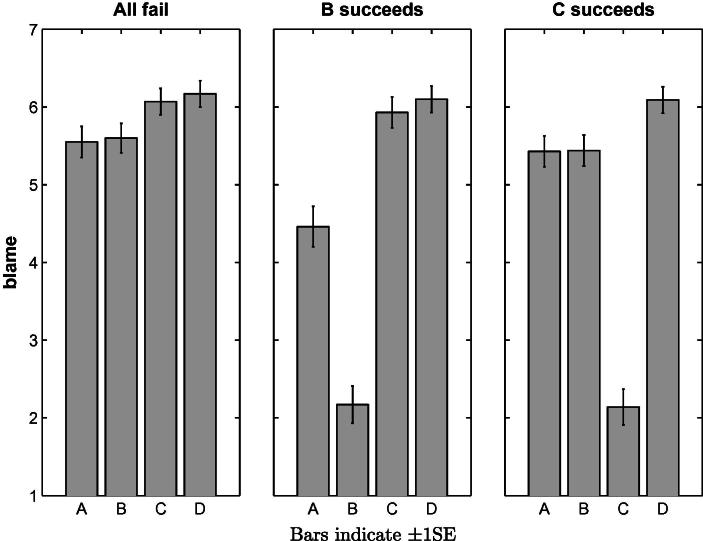
Mean blame attributions in Experiment 1 to the four players *A*, *B*, *C* and *D* for the situations in which *all fail*, *B succeeds* and *C succeeds*.

The blame attributions obtained for Questions 2–4 are presented in Fig. 3. In order to test whether participants’ blame attributions differed between the players and situations, we conducted a repeated-measures ANOVA with Player (*A*, *B*, *C* and *D*) and Situation (all failed, *B* succeeded, *C* succeeded) as within-subjects factors. We found main effects of Player, *F*(3, 204) = 63.60, *p* < .001, *partial η*^2^ = .483 and of Situation, *F*(2, 136) = 60.63, *p* < .001, *partial η*^2^ = .471 as well as an interaction effect, *F*(6, 408) = 76.34, *p* < .001, *partial η*^2^ = .529. Having established that participants’ blame attributions were influenced by our experimental manipulation, we proceed with a series of pairwise *t*-tests to test the more specific comparisons for which the models discussed above make different predictions.^6^

The blame attributed to *A* is affected by the individual outcomes of the other team members. Compared to the baseline when all team members fail, blame is decreased when *B* succeeds (5.55 vs. 4.46, *t*_(81)_ = 4.288, *p* < .001), thereby rejecting SimResp and SimPiv. Furthermore, the blame attributions depend not only on the number of team members who share the blame, but also on the causal relationships between them. *A*’s blame is higher when *C* succeeds compared to when *B* succeeds (5.43 vs. 4.46, *t*_(82)_ = 3.910, *p* < .001), thereby rejecting DiffResp. The best prediction is provided by CFPiv, although *A*’s blame does not increase when *C* succeeds compared to when all four fail, contrary to the prediction of the model (5.43 vs. 5.55, *t*_(81)_ = −.709, *p* = .480).

Another prediction of CFPiv not supported by the data is that all team members should receive the same blame when all fail. The minimal number of changes required for pivotality is identical (*N* = 2) for each player. For example, in order to render *A* pivotal, a counterfactual situation needs to be considered in which the values of *C* and *D* were changed from 0 to 1. Similarly, in order to render *C* pivotal, the values of *A* (or *B*) and *D* would need to be changed. However, players *C* and *D*, whose respective successes are necessary for a team win, are assigned more blame than players *A* and *B* (*F*_(3,79)_ = 4.981, *p* < .005). Furthermore, when *C* succeeds, *D* still receives more blame than *A* and *B* (6.09 vs. 5.43, *t*_(80)_ = 3.141, *p* < .005 and 5.44, *t*_(79)_ = 3.336, *p* < .005, respectively).

## 4 Experiment 2

In contrast to the prediction of the CFPiv model, the blame assigned to *A* did not increase as the number of changes required to achieve the counterfactual situation in which *A* is pivotal decreased. To see whether blame does increase in the extreme case in which the required number of changes is reduced to zero, we added a new situation to Experiment 2, in which both *C* and *D* succeeded in their individual tasks, thereby making *A* pivotal in the observed outcome. Additionally, we designed Experiment 2 to test the robustness of the results of Experiment 1 by repeating the same team challenge structure in a different framing, using computer interface, and with a different participant population.

### 4.1 Method

#### 4.1.1 Participants

Sixty-one psychology undergraduate students at University College London participated in the experiment as part of a lab exercise.

#### 4.1.2 Materials and procedure

We presented participants with the *dot-clicking game*, in which a dot is randomly repositioned on a computer screen each time the player clicks on it. The score in the game is defined to be the number of clicks made within an fixed duration of time. In the experiment, hypothetical players in a team play the dot-clicking game. Each player succeeds in her game if she obtains a given minimal score. The team outcome is determined by a combination of the individual outcomes, which was presented graphically to the participants as in Fig. 1. The structure of the game is equivalent to that used in Experiment 1. At the beginning of the experiment, participants played the dot-clicking game themselves to get a sense for the task. To avoid participants forming expectations based on their own performance, we stated that the game played by the hypothetical players was played for a different duration with a different-sized dot.

The first stage of the experiment was part of a separate study, and involved the participants making criticality attributions to players in different team challenges before the challenges are played. In the second stage of the experiment, participants used on-screen sliders to assign blame to the four players in the team challenge presented in Fig. 1, in response to the following question: “How blameworthy is each player for the team’s loss in this challenge?” The sliders corresponded to 11-point Likert scales (0 = ‘not at all’, 10 = ‘very much’). The four different outcome patterns were presented in random order.

### 4.2 Results and discussion

**Fig. 4:**
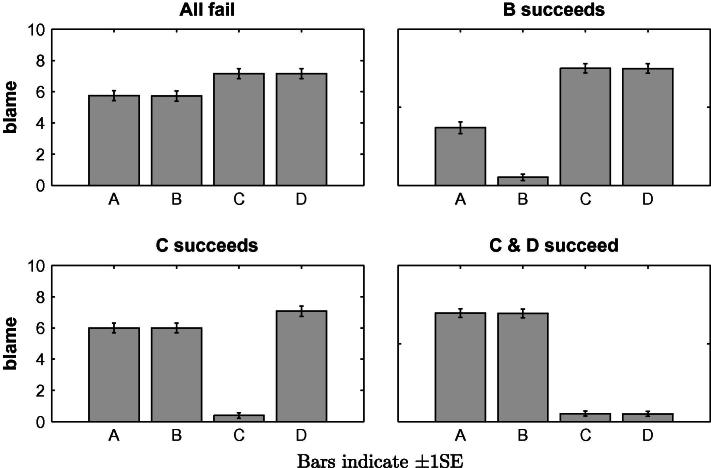
Mean blame attributions in Experiment 2 to the four players *A*, *B*, *C* and *D* for the situations in which *all fail*, *B succeeds*, *C succeeds* and *C and D succeed*.

The blame attributions are presented in Fig. 4. As in Experiment 1, a repeated-measures ANOVA with Player and Situation as within-subject factors revealed significant main effects of Player, *F*(3, 180) = 32.69, *p* < .001, *partial η*^2^ = .353 and of Situation, *F*(3, 180) = 58.58, *p* < .001, *partial η*^2^ = .494 as well as an interaction effect, *F*(9, 540) = 231.60, *p* < .001, *partial η*^2^ = .794. The patterns of blame attributions fully replicate those observed in Experiment 1. Compared to the baseline condition in which all players failed, the blame attributed to *A* significantly decreases when her substitute *B* succeeds (3.69 vs. 5.75, *t*_(60)_ = 4.972, *p* < .001), but does not significantly change when her complement *C* succeeds (6.00 vs. 5.75, *t*_(60)_ = 1.023, *p* = .310). However, when both complementary players *C* and *D* succeed, so that *A* becomes pivotal, *A* incurs significantly more blame (6.95 vs. 5.75, *t*_(60)_ = 3.687, *p* < .001). As in Experiment 1, if all four team members have failed their individual tasks, then players *C* and *D* are perceived as more to blame than *A* and *B* (*F*_(3,180)_ = 18.435, *p* < .001). Similarly, *D* is assigned more blame than both *A* and *B* when only *C* succeeds (7.07 vs. 6.00, *t*_(60)_ = 2.833, *p* = .006 for either comparison).

Taken together, the results of the two experiments establish that blame attributions made by our participants are sensitive to the causal structure. The highest blame is assigned to an agent in the situation in which she was pivotal, and the lowest blame is assigned when the most changes of individual outcomes are required in order to make the agent counterfactually pivotal. In contrast with diffusion of responsibility considerations, reducing the number of agents who share the blame has different effects when different agents’ outcomes are changed, and can even reduce the blame, depending on the causal structure and the relationship between the players. Thus, out of the four models we consider, the model of counterfactual pivotality provides the best explanation of the blame attributions observed in the experiments so far.

However, several findings remain unexplained by the model. In both experiments, the success of player *C* was not sufficient to increase the blame attributed to *A*, as predicted by the model, although the predicted effect was obtained in Experiment 2 when both *C* and *D* have succeeded. None of the theoretical considerations can explain the lack of effect in the former case, as it is predicted by both the CFPiv and DiffResp models.

More interesting is the systematic difference in blame between players *A* and *B* on one hand and *C* and *D* on the other hand within the same situation, when the minimal number of changes required to make an agent pivotal is the same for all those who failed in their individual task. We conjecture that this result is explained by the following observation. In the situation in which all of the team members have failed, the minimal change required to make each of them pivotal involves changing the outcomes of two other members. However, there is only one way to achieve this for team members *A* and *B*, namely by counterfactually changing the individual outcomes of *C* and *D*. In contrast, there are two ways to make each of *C* and *D* counterfactually pivotal, namely by changing the outcome of the other one as well as that of *either A* or *B*. For example, to make *D* counterfactually pivotal one must change *C* as well as either *A* or *B*. The same rationale holds for the situation in which only *C* succeeded. In this case, to make *D* counterfactually pivotal one must change either *A* or *B*, whereas to make *A* counterfactually pivotal one must change *D*.

This explanation implies that a minimal change model does not reflect the way in which people make responsibility attributions. Rather, when multiple paths exist in which an agent can be made counterfactually pivotal, blame increases accordingly. In the following sections we outline a model that expands the model based on Chockler and Halpern (2004) to include this insight and test the new model in a new experiment.

## 5 Multiple counterfactual pivotality

Consider the following situation: You are the manager of your home country’s team in the International Salsa Competition. Your team consists of Alice, Bob, Chuck and Dan. In order to compete in the tournament, Alice will need to show up and at least one of her partners. You instruct all of them to come to the tournament. However, as it turns out, none of them show up on the day of the competition. How much would you blame Alice for the fact that your team could not compete? How much would you blame Bob, Chuck or Dan?

The CFPiv model predicts that all of them will be blamed equally. Given that none of them showed up, a minimum of one change needs to be made in order to render Alice pivotal. We can either change Bob, Chuck or Dan from *not having showed* up to *having showed up*. Similarly, for Bob, only one change is needed to render him pivotal, namely changing Alice to having showed up. The same holds of course for Chuck and Dan. Hence, we see that all team members are predicted to receive equal blame. However, the intuition is strong that Alice carries more blame for the fact that the team could not compete than each of her partners, as there are more counterfactual situations in which her appearance is crucial for the team to compete.

In this section, we introduce a new model, which we term *multiple counterfactual pivotality* (MultCFPiv). The new model expands CFPiv to account for the results of Experiments 1 and 2. Recall that the CFPiv model assigns responsibility according to the minimal change required to attain pivotality. Our new model retains the principle of counterfactual pivotality, but allows for multiple counterfactual situations, in which an agent is pivotal, to be considered. The new model has three important features. First, adding new paths by which an agent can become pivotal increases her responsibility. Second, as in the CFPiv model, responsibility decreases with the number of changes required to attain pivotality along any single path. Lastly, the new model reduces to the CFPiv model if there is only one way in which the agent can become counterfactually pivotal.

In order to accommodate multiple paths to pivotality while maintaining the general framework specified by the CFPiv model, we define an *equivalent single path* for any situation in which multiple paths exist. A path in this context is simply defined as a series of changes to the individual outcomes of other team members required to turn the observed situation into a counterfactual situation in which the target agent is pivotal. The responsibility assigned to the agent in the multiple-paths situation is the same as that assigned by the CFPiv model with the equivalent single path. The number of changes, *N*, is defined to be 0 if the agent is already pivotal and otherwise

(1) $$N=\frac{1}{\sum_{i=1}^{k}\frac{1}{n_{i}}}\text{,}$$

where *k* is the number of different paths by which the agent can become pivotal, with required number of changes *n*_1_, *n*_2_, … , *n*_*k*_, respectively. The responsibility can then be defined to be 1/(*N* + 1), as in the original CFPiv model.^7^

It remains to define how the number and lengths of the multiple paths are determined based on the causal structure of the team challenge and the individual players’ outcomes. The first step is to identify all of the counterfactual outcome profiles in which an agent would be pivotal, and to determine the differences between each such counterfactual situation and the actually observed situation. Note that ordering the sequences sequentially defines a series of changes, or a path, that turns the actual situation into the counterfactual one. Next, exclude the situations for which the target agent is pivotal at an earlier step along one or more paths. In other words, a counterfactual situation is excluded if one of the changes made in order to attain it can be undone without eliminating pivotality. For each of the *k* remaining counterfactual situations, the minimal change (shortest path) is entered into the responsibility attribution.

To illustrate, consider the team challenge in Fig. 1. As noted earlier in the discussion of our experimental results, if all four team members have failed their individual tasks, it is possible to make any of them pivotal through two counterfactual changes. Accordingly, the blame assigned to any team member according to CFPiv is 1/(2 + 1) = 1/3. Nevertheless, for both *A* and *B*, there exists exactly one counterfactual situation in which they are pivotal, hence MultCFPiv also assigns them a blame of 1/3. Conversely, for either *C* or *D*, there are three counterfactual situations in which they are pivotal. Namely, when the other one succeeds, in addition to either *A*, *B*, or both *A* and *B*. Since any path to the latter situation (in which both *A* and *B* succeeded) must go through one of the first two (in which either *A* or *B* succeeded), we exclude it from the analysis. Thus, we end up with two paths by which pivotality can be reached, each involving two changes. The number of changes in the equivalent single path is given by $\frac{1}{\frac{1}{2}+\frac{1}{2}}=1$. Therefore, the responsibility assigned to *C* and *D* by our model is 1/(1 + 1) = 1/2.

**Fig. 5:**
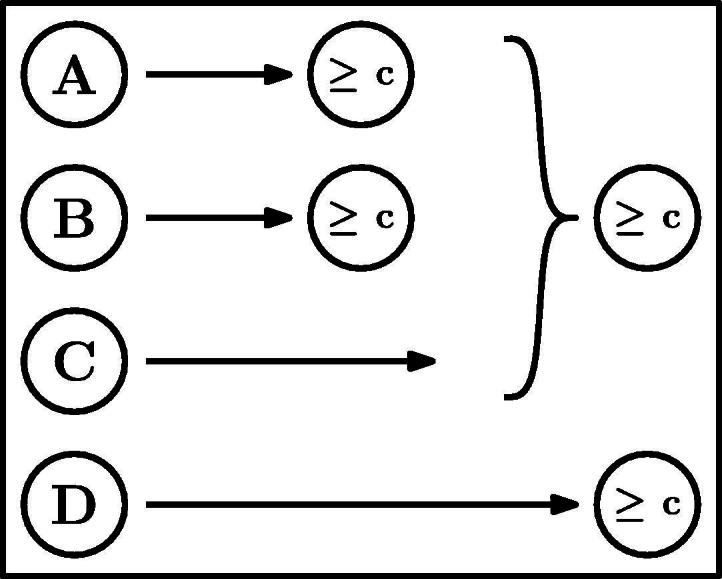
Team challenge in Experiment 3. For the team to win, either both *A* and *B*, or *C*, as well as *D* must win.

As a further illustration, consider the team challenge in Fig. 5, in which the team wins if *D* succeeds in addition to either *C* or both *A* and *B*, hence *t* = *min*(*max*(*o*_*A*_, *o*_*B*_), *o*_*C*_) × *o*_*D*_. Once more, assume that all four team members have failed in their individual tasks. In this case, the CFPiv model assigns a blame of 1/3 to *A* and *B* and 1/2 to *C* and *D*. For example, to make *A* pivotal we need to change *B* and *D*, so *N* = 2, to make *D* pivotal we need to just change *C*, so *N* = 1, and to make *C* pivotal we just need to change *D*, so *N* = 1. The predictions of MultCFPiv differ only with regard to team member *D*, who is pivotal in three counterfactual situations, namely when *C* succeeds, when *A* and *B* succeed or when *A*, *B* and *C* succeed. As in the previous example, the model does not consider the latter situation in which all three other team members have succeeded, since a subset of the changes is sufficient for pivotality. There remain two paths to pivotality. One is by changing the outcome of *C* (*n*_1_ = 1), the other is by changing the outcomes of both *A* and *B* (*n*_2_ = 2). The number of changes in the equivalent single path is now $\frac{1}{\frac{1}{1}+\frac{1}{2}}=\frac{2}{3}$, and the blame assigned to *D* is hence $1/\left(\frac{2}{3}+1\right)=0.6$. Experiment 3 tests the novel prediction that team member *D* incurs more blame than the other three in the team challenge of Fig. 5, in the case that all four team members failed their individual tasks.

## 6 Experiment 3

To test the novel predictions derived from the MultCFPiv model, we constructed the team challenge depicted in Fig. 5, in which the team wins if *D* succeeds in addition to either *C* or both *A* and *B*. The new challenge also serves as an additional test of the hypotheses tested in the previous experiments. We argued above that an implication of the CFPiv model is that how much blame a player incurs, reduces with each successful substitute and increases with each successful complement. The new team challenge provides a test for this generalization. In this challenge, player *A* is complementary to player *B*, and is a substitute of player *C* (in the case where *B* succeeds). As in the previous experiments, we start with a baseline situation in which all team members failed in their individual task, and compare blame attributions to player *A* when we reduce the number of failed team members. As in the team challenge of Fig. 1, the failure of player *D* ensures that none of the other players is pivotal.

**Fig. 6:**
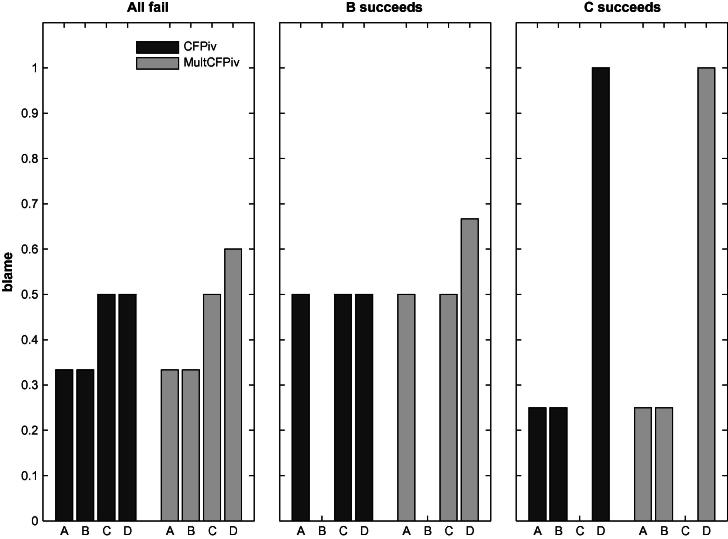
Blame attributions according to the Counterfactual Pivotality (CFPiv) and the Multiple Counterfactual Pivotality (MultCFPiv) models in Experiment 3.

Fig. 6 presents the blame attributions predicted by the CFPiv and MultCFPiv models for each player in each of the experimental conditions. A comparison of the bars in the figure reveals the qualitative predictions tested in the experiments. The basic prediction of both models is tested by comparing the blame assigned to player *A* in the three conditions, as in the previous experiments. Namely, *A* receives more blame if *B* succeeds, but less blame if *C* succeeds. The two models differ with regard to the blame attributed to player *D*. The prediction of the MultCFPiv model to be tested is that *D* is more to blame than *C* when all fail or *B* succeeds, although both can become pivotal through only one change. In addition, the success of *B* reduces the number of changes required to make *D* pivotal along the longer path, which is ignored in CFPiv, hence only MultCFPiv predicts a higher blame for *D* as a result.

### 6.1 Method

#### 6.1.1 Participants

Forty participants from the USA, 13 males and 27 females, ages 19–57 (mean 32) were recruited to participate in the experiment via Amazon Mechanical Turk for a flat fee of $1.

#### 6.1.2 Materials and procedure

The procedure was similar to that of Experiment 2. The team challenge used was the one depicted in Fig. 5, and the three conditions were (a) all fail, (b) *B* succeeds, and (c) *C* succeeds. Participants provided blame attributions on sliders corresponding to 21-point Likert scales.

### 6.2 Results and discussion

**Fig. 7:**
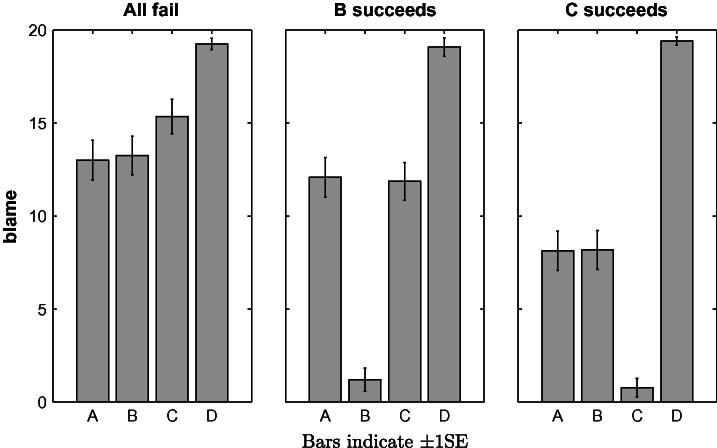
Mean blame attributions in Experiment 3 to the four players *A*, *B*, *C* and *D* for the situations in which *all fail*, *B succeeds* and *C succeeds*.

Generally, the patterns observed in the previous two experiments were replicated with the new team challenge (see Fig. 7). A repeated-measures ANOVA with Player and Situation as within-subject factors revealed significant main effects of Player, *F*(3, 234) = 89.52, *p* < .001, *partial η*^2^ = .534 and of Situation, *F*(2, 156) = 50.96, *p* <  .001, *partial η*^2^ = .395 as well as an interaction effect, *F*(6, 468) = 62.64, *p* < .001, *partial η*^2^ = .445. The blame assigned to player *A* significantly decreases if the substitute player *C* succeeds (8.13 vs. 13.00, *t*_(39)_ = 4.452, *p* < .001), but does not significantly differ if the complement player *B* succeeds (12.08 vs. 13.00, *t*_(39)_ = −1.045, *p* = .303). The effect of reducing the number of team members who failed significantly depends on the role of the team member who succeeded in the individual task, as player *A* receives more blame when *B* succeeds compared to when *C* succeeds (12.08 vs. 8.13, *t*_(39)_ = 3.209, *p* < .005).

The new challenge produces new test cases for the MultCFPiv model. In the situations where the CFPiv and MultCFPiv models diverge, the results are in line with MultCFPiv. When all of the four players fail, player *D* receives more blame than player *C* (19.25 vs. 15.35, *t*_(39)_ = 4.444, *p* < .001), who in turn receives more blame than player *A* (15.35 vs. 13.00, *t*_(39)_ = 3.116, *p* < .005) or player *B* (15.35 vs. 13.35, *t*_(39)_ = 2.723, *p* < .01). Similarly, player *D* is perceived as more blameworthy than player *C* when player *B* succeeds (19.08 vs. 11.87, *t*_(39)_ = 7.232, *p* < .001). However, the blame attributed to player *D* does not significantly change across situations, possibly due to a ceiling effect, as the average blame rating is above 19 out of 20 in all three situations.

The data yield one surprising result, which is not predicted by any of the models we consider. Player *C* is assigned less blame when player *B* succeeds compared to when all of the players fail (11.87 vs. 15.35, *t*_(39)_ = 3.575, *p* < .001). Note that the relationship between players *B* and *C* is one of substitution. Hence the finding, albeit not predicted by MultCFPiv, is consistent with the general principle implied by counterfactual pivotality reasoning which states that responsibility is reduced when a peer succeeds in the case of substitution.

In sum, the results of the new experiment are consistent with the results obtained with the previous team challenge. Out of the five models we consider, the model based on multiple counterfactual pivotality best explains the data. Although some differences predicted by the model are not apparent in the data, we take the results to confirm the basic role of counterfactual pivotality in blame attributions.

## 7 General discussion

This paper provides a simple and clear test for possible models designed to capture the way in which people make responsibility attributions in a team environment. The results of three experiments that varied the causal structure of the team environment are broadly in line with a model that considers not only whether the person under consideration was pivotal in the actual situation but also by how close the person was to making a difference in other counterfactual situations. Two general principles follow from counterfactual pivotality reasoning: First, blame attributions to an agent weakly *increase* with the number of successful peers in the case of complementarity, as the actual situation becomes more similar to one in which the agent is pivotal. Second, blame attributions weakly *decrease* with the number of successful peers in the case of substitution, as the actual situation becomes less similar to one in which the agent is pivotal.

These relationships are apparent in all of our experiments. The effect of a change in one team member’s performance on the blame incurred by her peer strongly depends on the way in which the respective contributions of the two interact with regard to the team outcome, in line with the theoretical predictions.

Our results enable us to extend the CFPiv model tested by Gerstenberg and Lagnado (2010). The CFPiv model only takes into account the minimal number of changes along a single path to render the person under consideration pivotal. In contrast, the MultCFPiv model is sensitive to how many paths there are to reach a counterfactual situation in which the person would be pivotal and how many changes to the actual situation would be required along each path.

The sensitivity to counterfactual causal reasoning implied by the experimental results can be interpreted in different ways. The number of paths and counterfactual changes at the heart of the model can be taken as mental steps or, alternatively, as reflecting the difficulty of bringing to mind a certain counterfactual state given the actual state. Thus, the model need not be taken as a literal process model, but as a support for the importance of counterfactual causal reasoning in responsibility attributions in group contexts.

The new model sheds light on responsibility attributions in a group setting. However, its applicability is more general. A substantial literature has developed over the last decades dealing with the way in which people make social attributions when there are multiple potential causes (Kun & Weiner, 1973; Leddo, Abelson, & Gross, 1984; McClure, Lalljee, Jaspars, & Abelson, 1989; Reeder & Brewer, 1979; Roese & Morris, 1999). This stream of the literature typically focuses on how people interpret a causally-ambiguous situation, often focusing on the relationship between internal causes, such as ability and effort, and external causes, such as task difficulty or luck (Kelley, 1972). Our extension of Chockler and Halpern’s (2004) model, when applied to intra-person causes rather than to group members, has the power to complement this literature by providing a framework for understanding how responsibility is attributed to multiple causes when the causal structure is unambiguous. One novel development of this approach would be to investigate responsibility attributions in situations where a single individual engages in a complex task with various subcomponents. For example, when a solo athlete competes in a multi-event game such as a decathlon, or when a chef must prepare all dishes himself.

The team environment studied in this paper was designed to capture the essentials of responsibility attributions in groups, and abstracts from many real-world features of team performance. We see several directions for future research to pursue in order to take into account important variables that are absent in our framework. In terms of the task structure, the current model is somewhat restricted by the dichotomous individual and team outcomes and deterministic integration function. Future models should be developed to account for probabilistic structures. These can include probabilistic processes at the level of performance, e.g., through expectations, and at the level of outcomes, by a probabilistic integration function.

The model is based on the causal structure of the team task, and as such abstracts from characteristics of the agents who are assigned responsibility. For example, the model can be equally applied to voluntary actions and to physical occurrences. However, factors such as intentionality and foreseeability have been shown to play an important role in responsibility attributions (Gerstenberg, Lagnado, & Kareev, 2010; Gerstenberg & Lagnado, 2012; Lagnado & Channon, 2008; McClure, Hilton, & Sutton, 2007; Schächtele, Gerstenberg, & Lagnado, 2010). A related issue is the choice element in the action. In our setup, the individual outcome is (assumed to be) determined by a combination of skill and luck. However, the model can be easily applied to strategic decision-making situations such as contributions to a threshold public good (Rapoport, 1987). Future work is needed to establish whether different cognitive rules govern responsibility attributions in strategic contexts.

In our theoretical analysis and empirical implementation, we did not treat responsibility and blame as separate constructs. However, several frameworks exist that distinguish between the two concepts based on various considerations. Compared to responsibility, blame is associated with negative, and in particular severe, outcomes (Weiner, 1995); can be mitigated by possible justifications (Shaver, 1985) and ignorance on the part of the acting agent (Chockler & Halpern, 2004); and in the view of some scholars is closely linked with emotional responses (Alicke, 2000). These distinctions are not consequential in our simple framework, in which we look at blame attributions in a somewhat artificial and impartial setting which is not likely to arouse strong emotional responses. Having established the role of counterfactual and causal reasoning in responsibility attributions in such minimal setting, further research can incorporate mitigating and affective aspects into the situation. Moreover, the framework can be naturally extended to include the epistemic states of the agents.

Finally, we studied responsibility attributions made by external observers, somewhat similar to attributions made by jury members or sports fans. In many relevant situations, however, responsibility attributions are made by the team members themselves. In such situations, judges may have a higher motivation to reach an informed judgment, enhancing counterfactual reasoning. On the other hand, self attributions might be susceptible to a self-serving bias (Campbell & Sedikides, 1999), shifting focus as to reduce blame or increase credit to oneself. Egocentric biases may also influence responsibility attributions by putting increased weights on counterfactuals involving oneself when attributing responsibility to peers.

Individuals make contributions to team projects across a large array of domains, ranging from school assignments to criminal activities. In many cases, those individuals incur blame or credit from themselves and from others. This paper establishes the important role of counterfactual and causal reasoning in responsibility attributions in teams, laying a foundation for the study of the way in which people place blame and credit within the rich environment of team performance.

## Figures and Tables

**Table 1:** Scenario and questions for Experiment 1.

In a new cooking show on television, a group of four chefs are charged with the task of preparing a meal in a certain culinary style. A meal is composed of two starters, one main dish and a dessert. The show panel judges each of the four dishes, and determines whether it is successful or not. The group wins the task if the meal is successful, i.e.
• At least one starter is successful • The main dish is successful • The dessert is successful

In other words, if there is a successful starter, a successful main dish, and a successful dessert, then the group wins even if one starter has failed. But if the main dish has failed or the dessert has failed, then the group has failed the task regardless of the success of the other dishes. The four chefs Oren, Benni, Gidi and Doron participate in one of the shows. After receiving their task, they decided to split the preparation between them so that each chef prepares one of the four dishes. The chefs did not agree on who will prepare which dish, so they decided to determine it by chance. It turned out that Oren prepares a starter, Benni prepares a starter, Gidi prepares the main dish, and Doron prepares the dessert
1. How much responsibility, do you think, does each of the chefs have for the success or failure of the task? 2. The show panel has tried the dishes and determined that none of the dishes was successful. Therefore the group has failed the task. To what extent, do you think, is each of the group members to blame for the group’s failure? 3. To what extent, do you think, would each of the group members be to blame had it been determined that Gidi’s main dish was successful, whereas the other three dishes were not? 4. To what extent, do you think, would each of the group members be to blame had it been determined that Benni’s starter was successful, whereas the other three dishes were not?

## Footnotes

1 Chockler and Halpern (2004) distinguish between responsibility and blame. Blame is defined as anticipated responsibility and thus relative to the epistemic state of the agent. However, since we do not vary epistemic states in our experiments, we will follow Gerstenberg and Lagnado (2010) in equating blame with negative responsibility (see Robbennolt, 2000, for a discussion of the different response measures).

2 Chockler and Halpern (2004) define *N* in terms of the number of variables in the causal model whose value needs to be changed. It would of course also be possible to consider changes to the causal model, for example by changing the team function *f*. However, while there are many possible ways in which the causal model could be changed, the notion of change in terms of the number of variables is well-defined. Hence, to allow for a clear test of the model, we restrict our analysis to the simple and intuitive changes to variables *within a given causal model*.

3 Note that counterfactual reasoning is already required to ascertain actual (simple) pivotality. Counterfactual pivotality thus involves two levels of counterfactual reasoning – thinking about counterfactual worlds in which the outcome could be counterfactually altered by the individual.

4 The original Hebrew version is available upon request. Demos of Experiments 2 and 3 can be accessed here: http://www.ucl.ac.uk/lagnado-lab/experiments/demos/finding_fault/finding_fault.html.

5 Question 1 was included as part of a separate research program, and is not analyzed in the current paper.

6 Somewhat surprisingly, the average blame attributed to players who succeeded in their individual tasks was higher than the minimal possible value of 1. We attribute this result to a number of unmotivated participants who provided random responses (e.g., marking the same value throughout the form). This behavior is close to absent in the better-controlled Experiment 2, which replicates all the qualitative results of the current experiment.

7 This definition relies on the harmonic mean of the number of changes, and mirrors similar equivalencies in physical systems such as hydraulics and electricity. For example, it is isomorphic to the resistance in an electric circuit, in which each change is represented by a resistor of 1Ω, and resistors are connected serially to represent the number of changes along a path, and in parallel to represent multiple paths. We thank Yaniv Edery for suggesting this analogy.
